# The Triterpenes 3β-Lup-20(29)-en-3-ol and 3β-Lup-20(29)-en-3-yl Acetate and the Carbohydrate 1,2,3,4,5,6-Hexa-*O*-acetyl-dulcitol as Photosynthesis Light Reactions Inhibitors

**DOI:** 10.3390/molecules16129939

**Published:** 2011-12-01

**Authors:** Djalma Menezes-de-Oliveira, Maria-Isabel Aguilar, Beatriz King-Díaz, Sidney Augusto Vieira-Filho, Lucinier Pains-Duarte, Grácia-Divin de Fátima Silva, Blas Lotina-Hennsen

**Affiliations:** 1 de Química, Universidad Federal de Minas Gerais, Avenida Antonio Carlos 6627, Pampulha, 31270-901, Belo Horizonte, MG, Brazil; 2 de Bioquímica, Facultad de Química, Universidad Nacional Autónoma de México, México D. F. 04510, Mexico

**Keywords:** chlorophyll *a* fluorescence, energy transfer inhibitor, 1,2,3,4,5,6-hexa-*O*-acetyldulcitol, Hill reaction inhibitors, 3β-lup-20(29)-en-3-yl acetate, lupeol, lup-20(29)-en-3β-ol, *Maytenus acanthophylla*, uncoupler, *Xylosma flexuosa*

## Abstract

Three compounds were isolated from *Maytenus acanthophylla* Reissek (Celastraceae): the pentacyclic triterpenes lup-20(29)-en-3*β*-ol (lupeol, **1**) and 3*β*-lup-20(29)-en-3-yl acetate (**2**) and the carbohydrate 1,2,3,4,5,6-hexa-*O*-acetyldulcitol (**3**);lupeol was also isolated from *Xylosma flexuosa*. The compounds’ structures were elucidated by spectroscopic and spectrometric analysis. Compound **1** acts as an energy transfer inhibitor, interacting with isolated CF_1_ bound to thylakoid membrane, and dulcitol hexaacetate **3** behaves as a Hill reaction inhibitor and as an uncoupler, as determined by polarography. Chlorophyll *a* (Chl *a*) fluorescence induction kinetics from the minimum yield F_0_ to the maximum yield F_M _ provides information of the filling up from electrons coming from water to plastoquinone pool with reducing equivalents. In this paper we have examined the effects of compounds **1** and **3** on spinach leaf discs. Compound **1** induces the appearance of a K-band, which indicates that it inhibits the water splitting enzyme. *In vivo* assays measuring the fluorescence of chl *a* in *P. ixocarpa* leaves sprayed with compound **1**, showed the appearance of the K-band and the PSII reaction centers was transformed to “heat sinks” or silent reaction centers unable to reduce Q_A_. However, **3** also induced the appearance of a K band and a new band I appears in *P. ixocarpa* plants, therefore it inhibits at the water splitting enzyme complex and at the PQH_2_ site on b_6_f complex. Compounds **1** and **3** did not affect chlorophyll *a* fluorescence of *L. perenne* plants.

## 1. Introduction

*Maytenus acanthophylla* Reissek (Celastraceae) is a medicinal plant found in Bahia state, Brazil [1] where it is vulnerable to extinction and the sp. is already considered extinct in Minas Gerais [2]. Its chemical constituents isolated from roots include gutta-percha, flavonoids, alditol, lupanes, oleanane, ursane, and quinonamethides [3]. *Xylosma flexuosa* (Flacourtiaceae) is a tree or sometimes a shrub distributed in Mexico, Guatemala, Honduras, El Salvador, Nicaragua, Costa Rica, Panamá, Venezuela and Curaçao [4]. In Mexico it is vulnerable to extinction. The chemical constituents of the methanol extract of *Xylosma flexuosa* are (*rel*)-2-([2,6-dibenzoyl]-β-glucopyranosyloxy)-5-hydroxybenzoyl-1*R*,2*R*,6*R*-trihydroxy-3-oxocyclohex-4-enoate (xilosmin) and three glycosides: Salirespolide, poliotrisoside and 2′-benzoylpoliotrisoside [5].

**Figure 1:**
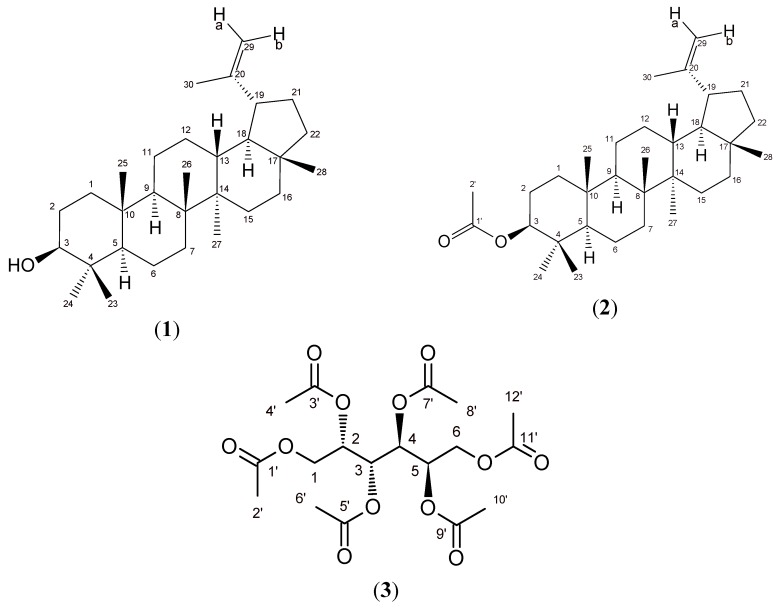
Structures of lup-20(29)-en-3β-ol (lupeol, **1**), 3 β-lup-20(29)-en-3-yl acetate (**2**) and 1,2,3,4,5,6-hexa-*O*-acetyldulcitol (**3**).

As part of our study of bioactive metabolites from species of the Flacourtiaceae and Celastraceae plant families, the present investigation describes the isolation, identification, and photosynthetic inhibitory activities of two triterpenes: lupeol, 3β-lup-20(29)-en-3-ol (**1**), 3β-lup-20(29)-en-3-yl acetate (**2**) and the carbohydrate 1,2,3,4,5,6-hexa-*O*-acetyldulcitol (**3**) (Figure 1) obtained from *M. acanthophylla*; compound **1** was also isolated from *X. flexuosa*. Although a relatively large number of highly phytotoxic allelochemicals are derived from the terpenoid pathway [6,7], the mode of action of some terpenoids indicate that they participate in plant-plant interactions, and few of them have been found to affect photosynthesis [8,9]. Chlorophyll a fluorescence kinetics of photosystem II analysis indicated that the primary target of synthetic herbicides is the photosynthetic apparatus on the acceptor side of photosystem II (PSII), by displacing the quinone Q_B_ from the D_1_ protein demonstrated by Velthuys [10]. Furthermore, most of the natural products that affect photosynthesis have diverse targets of action on chloroplasts electron transport chain [11]. Therefore, our aim was to study the effect of these two triterpenes and the acetylated-alditol as natural photosynthetic inhibitors by fluorescence of chlorophyll *a* activity that could suggest their participation in plant-plant interactions.

## 2. Results and Discussion

### 2.1. Effect of Lupeol (***1***), 3β-Lup-20(29)-en-3-yl Acetate (***2***) and 1,2,3,4,5,6-Hexa-O-acetyl-dulcitol (***3***) on Photophosphorylation

Compounds **1**–**3** were monitored by assay the photophosphorylation inhibition of the water to methylviologen (MV) step in freshly lysed intact chloroplasts. Lupeol (**1**) and dulcitol hexaacetate **3** were found to be the bioactive compounds and this activity was displayed in a concentration dependent-manner, with I_50_ values of 123 and 143 μM, respectively. The I_50_ values were extrapolated from the plot of the rate of ATP formation against increasing concentration of compounds. 3β-acetyl lupeol **2** showed less inhibitory activity than compounds **1** and **3** and, its I_50_ value could not be estimated, thus compound **2** were not further studied. These results suggested that the 3β-ol moiety on lupeol is important for its activity, since when it was acetylated this suppressed drastically its inhibitory activity.

### 2.2. The Effect of Compounds ***1*** and ***3*** on Different Photosynthetic Activities

Further investigation of compounds **1** and **3** as potential natural inhibitors of electron flow, their photosynthetic inhibitory effects on non-cyclic electron transport on spinach chloroplasts from water to MV was evaluated under basal, phosphorylation, and uncoupled conditions previously incubated for 1 min. Lupeol (**1**) inhibited basal and phosphorylating electron transport by 20% at 300 µM and the uncoupled electron transport (Table 1) was not affected. However when the chloroplasts was incubated with the compound **1** for 7 min, the basal and phosphorylating electron flow inhibition increased by 100% and 73%, respectively, and uncoupled electron flow was not affected (Table 1).

The main distinguishing features of most energy transfer inhibitors decrease the rate of electron flow only under conditions where active phosphorylation can take place, and electron flow in the absence of Pi (basal electron flow) or in the presence of uncouplers is either not affected or only slightly inhibited, with some exceptions, *i.e.*, Dio-9 which also inhibits basal electron flow [12], therefore, lupeol acts as a mild energy transfer inhibitor similar to Dio-9.

**Table 1:** Effect of 300 µM lupeol on the electron transport rate (basal, phosphorylating and uncoupled) measured from H_2_O to MV in isolated chloroplast was incubated for 1 and 7 min. The data are the average of three replicates.

Time (min)	Conc. (µM)	Basal		Phosphorylating		Uncoupled	
		*a*	*b*	*a*	*b*	*a*	*b*
1	0	624	100	835	100	930	100
	300	499	80	710	85	930	100
7	0	334	100	667	100	1480	100
	300	0	0	180	27	1401	95

*a* = µequiv. e^−^/mg Chl × h; b = %.

Furthermore, when energy transfer inhibitors are added to the thylakoid membrane bound Mg^2+^-ATPase or trypsin treated CF1 they inhibit the activity, with the exception of kaempferol [12] which had little effect on membrane bound Mg^2+^-ATPase. Moreover, isolated CF1 also catalyzes the Mg^2+^-ATPase or a rapid Ca^2+^-dependent ATPase reaction [12], the activity may be inhibited or enhanced by energy transfer inhibitors. Lupeol (100 μM) inhibited the membrane bound Mg^2+^-ATPase activity by 35% (Table 2) and thereafter its inhibitory effect decreased and the isolated CF_1_ Ca^2+^-ATPase activity was enhanced by 78% with lupeol (**1**, 300 μM) and it had no effect on the isolated Mg^2+^-ATPase activity (data not shown); these results indicate that lupeol interacts with isolated CF1 or when CF1 is bound to thylakoid membranes. McCarthy [12] concluded on the action of energy transfer inhibitor that “since all of these activities were shown to be catalyzed by the same enzyme (CF1), it appears that the chloroplast membrane confers special properties on CF1” in accord with our result, compound **1** enhanced Ca_2_^+^-ATPase activity in isolated CF1 and inhibits the Mg_2_^+^-ATPase activity bound to thylakoids membrane. We found that this enzyme is also targeted by others natural products like 2-methoxyethyl-7β-hydroxy-6-oxovouacapan-17β-oate and 3-methylbut-2-enyl-7β-hydroxy-6-oxovouacapan-17β-oate [8], epifriedelinol and canophyllol [9], as well as by other compounds like Dio-9, phlorizin, tri-*n*-butyltinchloride [12]. Ammonium chloride was used as positive control.

**Table 2:** Effect of lupenol on Mg^2+^-ATPase activity bound to thylakoid membrane and on the isolated CF_1_-Ca^2+^-ATPase activated by light and heat, respectively. Data are average of three replicates.

Lup-20(29)-in-3β-ol [μM]	Mg^2+^-ATPase (%)	Ca^2+^-ATPase (%)
Control	100	100
100	65	182
200	86	178.3
300	98	178.8

**Figure 2:**
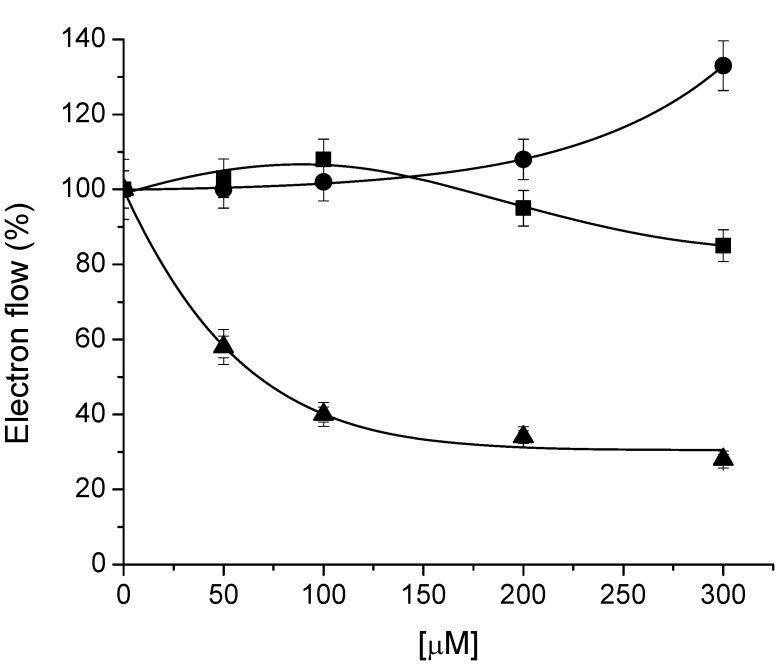
Effect of **3** on electron flow from water to MV in spinach chloroplasts on three different conditions: basal (■), phosphorylating (●), and uncoupled (▲) rate on isolated spinach chloroplasts. Control rate values were 624, 930 and 835, respectively in μequiv. e^−^ mg^−1^ Chl h^−1^. Other conditions are indicated in the Experimental section. Data are average of three replicates.

Compound **3** inhibited the uncoupled electron transport as its concentration increased up to 300 μM with an I_50_ value of 67 µM, furthermore, compound **3** (300 µM) enhanced the phosphorylating electron transport rate by 30% (Figure 2). The whole results on electron flow indicate that 1,2,3,4,5,6-hexa-*O*-acetyldulcitol (**3**) acts as Hill reaction inhibitor and as a mild uncoupler when the thylakoids were incubated with compound **3**.

### 2.3. Chl a Fluorescence Measurements in Spinach Leaf Discs with and without Compounds ***1*** and ***3***

**Figure 3:**
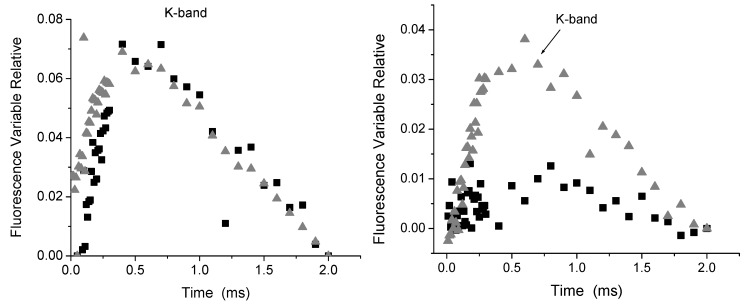
Appearance of K bands from fluorescence kinetics (F_t_) expressed as the kinetics of relative variable fluorescence of chlorophyll *a* on the spinach leaf disk incubated with two different concentrations 150 µM (■), 300 µM (▲) of compound **1** (left, panel A) and compound **3** (right, panel B). Data are average of five replicates.

Any photosynthetic sample in any physiological state exhibits upon illumination a fast fluorescence rise leading to a transient *F*_0_-K-J-I-P from initial fluorescence intensity *F*_0_ to a maximal intensity *F*_P_ or *F*_M_ with saturating light intensity. Between these two extremes the fluorescence intensity *Ft* shows intermediate steps as follows: *F*_J_ appears at about 2 ms; *F*_I_ appears at about 30–50 ms; and *F*_M_ appears at about 300–500 ms; K-band appears at about 300 μs in heat-stressed samples [13,14] or in the influences of chemicals or secondary metabolites [8,9,15,16,17]. Strasser [18] concluded that the appearance of the K-band is satisfactorily explained by an imbalance between the electron flow to the acceptor side and the electron flow from the donor side leading to the accumulation of Y_Z_^+^. Thus, any treatment or stress condition which affects the donor side capacity will make the K-band apparent, if the electron flow to the acceptor side is sufficient. Therefore, the K-band can be used as a specific indicator of injury to the OEC. Here Chl *a* fluorescence induction kinetic curve on spinach leaf discs incubated with compounds **1** and **3** for 12 h in the dark was measured in order to localize the target of the compounds on the electron transport chain. Control spinach leaf discs showed a typical polyphasic rise (OJIP curve) [10]. The plot on a logarithmic time scale revealed that there are large differences between the control and samples treated with **1** or **3** 150 µM and 300 µM changed to polyphasic rise called OKJIP curve (data not shown). Therefore, the OJIP kinetics data was normalized between F_J_ and F_0_, and expressed as V_t_ = (F_t_ − F_0_)/(F_J_ − F_0_) and interpreted as a measure of the fraction of the electron acceptor Q_A_ being in its reduced state [13,15]. The results show a fast rise at about 300 μs thus appears the K-band (Figure 3A). Thus, compound **1** inhibits the water splitting enzyme at the oxygen-evolving complex (OEC).

**Figure 4:**
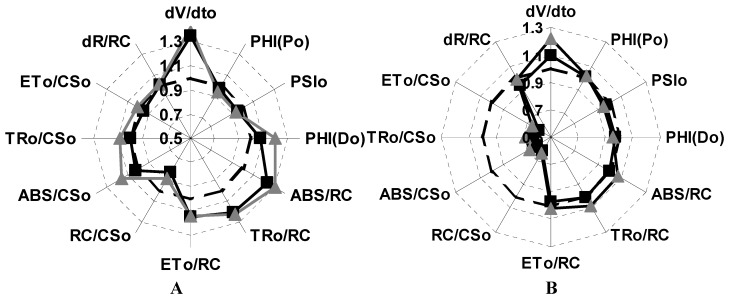
Radar plot graphs show the effects of compound **1** (Panel A) and compound **3** (panel B) on chlorophyll *a* fluorescence parameter calculated from OJIP curve. Both compounds were infiltrated on spinach leaf disk at three different concentrations 150 µM (■) and 300 µM (▲) respectively, and compared with the control leaf disc (dashed black line). The incubation time with the compounds for each treatment was 12 h and then adapted in the dark for 30 min.

The OJIP kinetics data was also normalized to a intermediate steps I and F_0_, or P and F_0_, and various parameters was calculated and the data were plotted as a radar graph, a circular graphic with a series of spokes or rays projecting from a central point, with each ray label representing a different variables (Figure 4). Figure 4A shows that lupeol (**1**), 150 and 300 µM, enhanced 35% (dV/dt_0_) (Figure 4A). (dV/dt_0_) is an approximate initial slope (in ms^−1^) of the fluorescence transient V = f(t) and is related to TR_0_ /RC which expresses the rate, per RC, by which excitons are trapped by RC resulting in the reduction of Q_A_ to Q_A_^−^ and calculated as = 4(F_300_ − F_0_)/(F_M_ − F_0_) which measure the rate of primary photochemistry ((dQ_A_^−^/Q_A(total)_)/dt_0_), where (dQ_A_^−^/Q_A(total)_ is the fraction of closed reaction center, therefore compound **1** make to function better the primary photochemistry.

When 300 µM of compound **1** was tested **it** enhanced by 30% the following parameters: the (ABS/RC) absorption flux per reaction center and the (TR_0_/RC) trapped energy flux per RC. Therefore, compound **1**, 300 µM induced a better light absorption through the RC and trapping of the electrons in the RC of PSII (ABS/RC), thus the pigment proteins and the RC have better thylakoid membrane functions due to conformation changes and compound **1** enhanced the activity. Finally, the following parameters: trapped energy flux per cross section (TR_0_/CS_0_); electron transport per reaction center (ET_0_/RC); quantum yield of energy dissipation, PHI(D_0_); the efficiency for an electron moves from the reduced intersystem electron acceptors to the end of PSI electron acceptors, dR/RC; the maximum quantum yield of primary photochemistry, PHI(P0); the probability that a trapped exciton moves an electron into the electron transport chain beyond Q_A_- and the electron transport per cross section (ET_0_/CS) were not affected with compound **1** (300 µM), indicating that either it had no-inhibition effect on the PSI electron transport chain or had a non-significant value. Moreover, the phenomenological fluxes RC/CS_0_ decreased 20% with compound **1** (300 µM), which indicates that a % of energy flux is dissipated as heat. We conclude that lupeol (**1**) partially inhibits and interacts with the water splitting enzyme and partially with the Mg_2_^+^-ATPase bound to thylakoid membrane and interacts also with the PSII RC by changing the architecture of the thylakoid membrane and affects the energy migration properties by enhancing its function.

When the spinach leaf discs were treated with **3** (150 and 300 µM), the following phenomenological energy fluxes per excited cross section ABS/CS_0_, TR_0_/CS, and ET_0_/CS were reduced by 50% with both concentrations; these results indicate that PSII electron transport chain and pigment protein were damaged with compound **3**, moreover, (dV/dt_0_) function was increased by 10 and 30%, respectively, with both concentrations, indicating that the PSII electron flow function worked with major efficiency. Other fluorescence parameters were not or slightly affected (Figure 4B). The parameters calculated from the fluorescence kinetic profiles showed an additional rapid appearance of the K-band at about 300 µs range (Figure 3B) induced with compound **3**, therefore, its target is the water splitting enzyme which in normal conditions donates electrons to PSII by a reduction of its activity. This site is also targeted by other natural products such as pachypodol [11], labdane-8α,15-diol [16] and 1-*O*-acetyl-12,13-dihydroxanthorrhizol [17].

**Figure 5:**
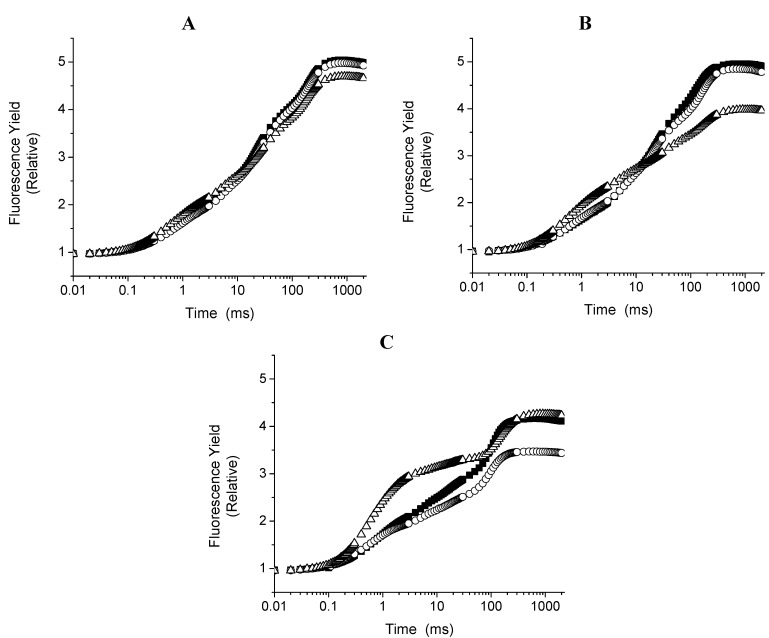
Curves OJIP measured in leaf discs affected after 30 min. (panel A), 4 h (panel B) and 12 h (Panel C) of incubation with 300 µM lupeol (○) and 50 µM DCMU (△). Control (■). The data are the average of ten replicates.

The results presented here allow us to conclude that **1** and **3** interact and inhibit the donor side of PSII in spinach leaf discs previously incubated for 12 h in the dark. The major effect of **1** and **3** on leaf disc fluorescence of chlorophyll a transient rather than in thylakoids indicates that these compounds are not metabolized and all of them might reach the target within the 12 h. This observations are supported due to the fact when the discs leaf were incubated for 30 min and 4 h with 300 µM of lupeol, or 50 µM of DCMU, in both cases they reached partially the inhibition site (see Figure 5), furthermore, with polarography studies using the thylakoids incubated with compounds by **1** (300 µM) and 7 min, the results show (Table 1) a major inhibition was at 7 min. suggesting that compounds **1** and **3** have difficulties to reach their targets, thus require more incubation to totally affect thylakoids’ light reaction activities.

### 2.4. In Vivo Assays

**Figure 6:**
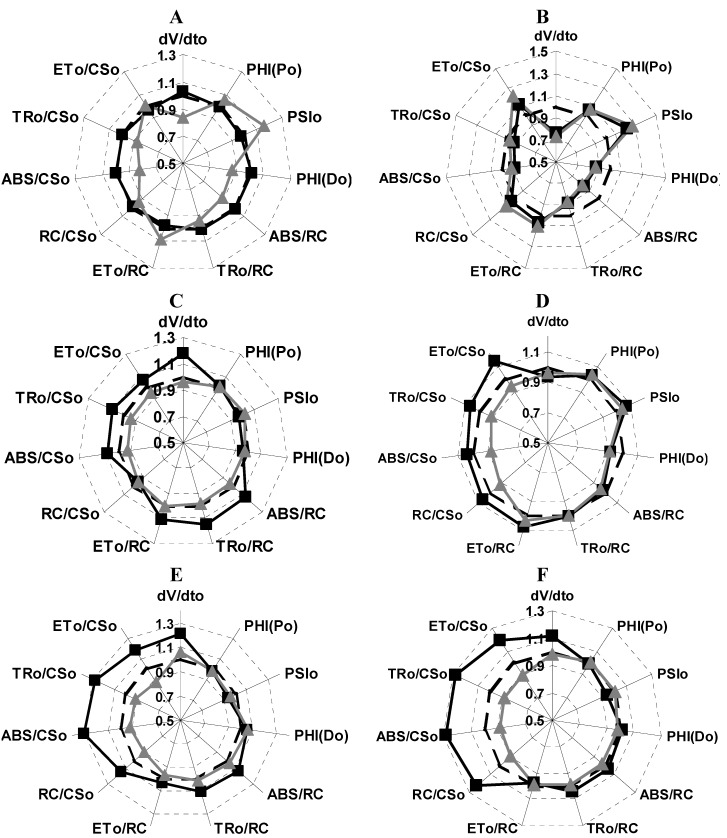
Radar plot showed the effect of compounds **1** and **3** on different calculated parameters from OJIP curves measured on *Physalis ixocarapa* plants treated with **1** after 24 h (Panel A), 48 h (Panel C) and 72 h (Panel E) at two different concentrations of **1**, 150 µM (■) and 300 µM (▲). Panels B, D and F showed the effect of **3** measured under the same conditions as **1**.

To investigate if **1** and **3** inhibit plant growth and if they were metabolized *in vivo*, their effect on intact plant leaves of *P. ixocarpa* and *L. perenne* was tested using the fluorescence of Chl *a* technique. *P. ixocarpa* and *L. perenne*,dicot- and -monocot plants, were used to distinguish if the compounds have selectivity in inhibiting plant growth, due to differences in wax content, wall cell thickness, chemical composition or enzyme contents. The Chl *a* fluorescence transient were measured at 24, 48 and 72 h after treatment of leaves and compared with control. Compound **1** (150 µM) had no effect on fluorescence of Chl *a* kinetics at 24 h of treatment on *P. ixocarpa* plants, (Figure 6, Panel A). However, compound **1** (300 µM) caused a reduction in the absorption per reaction center (ABS/RC) by 9%, the absorption per cross section (ABS/CS_0_) by 15%, the trapping flux per cross section (TR_0_/CS) by 12% and PHI(D_0_) by 11%, but these results had non-significant values. When *P. ixocarpa* plants were treated for 48 h with compound **1** (300 µM) did not show any effect (Figure 6C). When we used **1** (150 µM) the dV/dt_0_ parameter was enhanced by 20%, indicating that PSII electron flow functioned with major efficiency (Figure 6C). Other fluorescence parameters were not affected or were slightly affected with non-significant values like ABS/RC by 10%, ET_0_/RC by 10%, TR_0_/RC by 10%, and cross section by 10 %. After treatment of *P. ixocarpa* plants with compound **1** (150 µM) for 72 h the following parameters increased by 30%: RC/CS_0_, ABS/CS_0_, TR_0_/CS_0_, ET_0_/CS_0_ (Figure 6, Panel E). The phenomenological fluxes are calculated as RC/CS_0_ = (ABS/CS_0_)/(ABS/RC) and with compound **1**, 150 µM indicated that the energy flux function with major efficiency. The ET_0_/CS_0_ (calculated as = [1 − (F_0_/F_M_)](1 − V_J_)F_0_) is the electron transport flux per cross section function with better efficiency in PSII electron flow.

**Figure 7:**
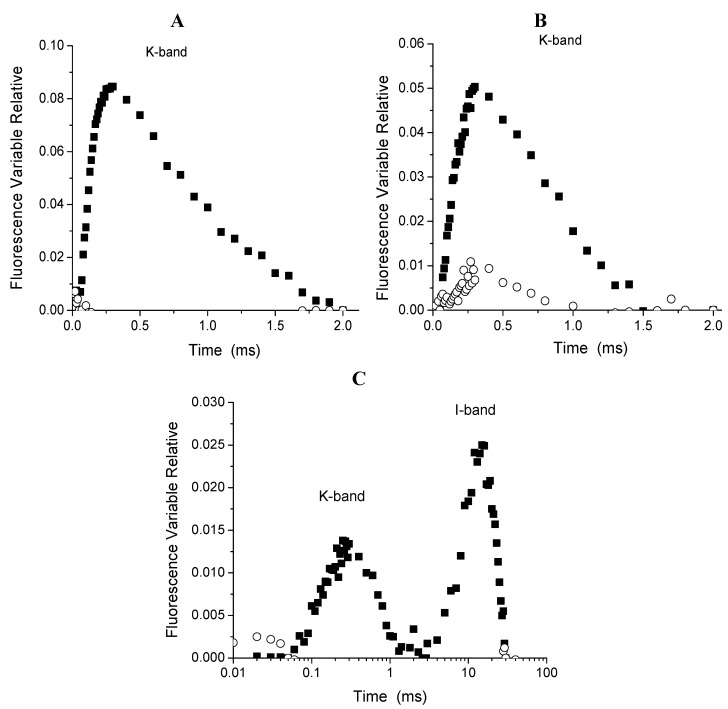
Appearance of K-bands at 48 h (Panel A), and 72 h (panel B) on fluorescence of chlorophyll *a* of *P: ixocarpa* plants treated with 150 µM (■) and 300 µM (○) of (**1**). Panel C shows the appearance of K and I bans after 72 h of treatments with **3**, 150 µM on *P. ixocarpa* plants.

When the parameter V_t_ = (F_t_ − F_0_)/(F_J_ − F_0_) was calculated it showed a faster rise of the appearance of the K-band at about 300 μs (Figure 7A), which indicates that compound **1** (150 µM) inhibits the water splitting enzyme at the oxygen-evolving complex (OEC). Furthermore, at 72 h of treatment with **1** (300 µM) the appearance a greater K-band was more noticeable (Figure 7B).

When *P. ixocarpa* plants were sprayed with compound **3** (150 and 300 μM), after 24 h of treatment the show some similar behavior as when treated with **1**. The following parameters PHI(D_0_), ABS/RC, TR_0_/RC decreased quasi 11% and dV/dt_0_ by 25%. However, parameters such as ET_0_/RC, RC/CS_0_ and ET_0_/CS increased by 10% and PSI_0_ by 30% (Figure 6B). Moreover, at 48 h of treatment of *P. ixocarpa* plants with **3** (150 μM) some parameters values were increased more: ET_0_/RC, RC/CS_0_ and ET_0_/CS and with compound **3** (300 μM) the same parameters decreased. Other parameters also increased slightly: ABS/CS_0_, TR_0_/CS_0_and PHI(P_0_) (Figure 6D), and at 72 h of treatment with **3**, 150 μM dV/dt_0_ was enhanced by 12% (Figure 6C) and it induced the appearance of the K and I bands (Figure 7, Panel C). These results confirm its inhibitor behavior at the water splitting enzyme as a target and a new interaction site appears the I-band around 20–30 ms. Oukarroum *et al.* [18] suggested that the appearance of an I-band is due to the heterogeneity of components such as Q_A_ and Q_B_ during the filling up the plastoquinone pool. Therefore, the target of compound **3** was located where PQH_2_ interact at its site on the b_6_f complex. This last site is also targeted by the natural product β-photogedunin acetate [19]. Chl *a* transients of *L. perenne* plants treated with **1**, and **3** at 150 and 300 µM were not affected after 24, 48 and 72 h (data not shown). These results indicate that compounds **1** and **3** were selective to *P. ixocarpa* plants.

## 3. Experimental

### 3.1. General

#### 3.1.1. Reagents

**Table:** 

Adenosine diphosphate	Adenosine triphosphate	Ascorbic acid
CaCl_2_	Chloroform	Ethyl acetate
2,5-Dibromo-6-isopropyl-3-methyl-1,4-benzoquinone	2,5-Dichloro-1,4-benzoquinone	2,6-Dichlorophenolindophenol
3-(3,4-Dichlorophenyl)-1,1-dimethylurea	Dimethyl sulfoxide (DMSO)	Dithiothreitol (DTT)
Ethylenediaminetetraacetic acid (EDTA)	*n*-Hexane	*N*-[2-Hydroxyethyl]piperazine-*N′*-[2-ethanesulfonic acid]
Isopropyl alcohol	KCl	KOH
MgCl_2_	Methanol	Methylviologen
NaCl	NaHCO_3_	Na_2_SO_4_
2-(*N*-Morpholino)-ethanesulfonic acid	Polyoxyethylensorbitan monolaurate (Tween-20)	Silica-gel 60-230 mesh
Silica-gel HF 60	Sodium silicomolybdate (SiMo)	Sorbitol
Sucrose	Trichloacetic acid (TCA)	Tricine

#### 3.1.2. Equipment

**Table:** 

Bruker DRX 400 (or 200) spectrometer for NMR experiments
Corning potentiometer model 12 (Corning Medical, Acton, MA, USA)
Gilson recorder (Kipp & Zonen, Bohemia, NY, USA)
Hansatech Fluorescence Handy PEA (plant efficiency analyzer)
Oxygraph YSI (Yellow Spring Instrument) Model 5300
Shimadzu QP-5050A Gas chromatography/Mass Spectrometry GC/MS, equipped with a PTE™-5 column (30 m, 0.25 mm, 0.25 µm, Supelco, USA)
Shimadzu QP5050A device for electron impact (70 eV) low-resolution mass spectra (EI-MS)

#### 3.1.3. Methods

##### 3.1.3.1. Plant Material

The leaves of *M. acanthophylla* were collected in April 2006 at Pé de Serra, Maracás, Bahia, Brazil. A voucher specimen (Collection No. OUPR-15532) was deposited in Herbário Professor José Badini, UFOP, Ouro Preto, Minas Gerais, Brazil. The leaves of *Xylosma flexuosa* (Flacourtiaceae) were collected in Zochipala-Leonardo Bravo, Guerrero, Mexico. A voucher specimen (SCME 056775) was deposited in the Herbario of Facultad de Ciencias, UNAM.

##### 3.1.3.2. Extraction and Isolation from *M. acanthophylla*

The air-dried leaves of *M. acanthophylla* were ground to a powder (2.8 kg) and extracted by percolation for five days with five liters of *n*-hexane (followed by ethyl acetate and methanol, five liters each for three days); the organic soluble fractions were concentrated under reduced pressure and tested. The bioactivity was located in the hexane (39.0 g) and methanol (55.0 g) extracts. Samples of the hexane extract (20.5 g) were subjected to silica gel column chromatography (CC) eluted with mixtures of *n*-hexane-chloroform of increasing polarity to afford nine fractions. Fraction (F5) collected with *n*-hexane-chloroform (3:7 v/v) was purified twice by silica gel flash CC eluted with *n*-hexane-chloroform-isopropyl alcohol (90:9:1 v/v/v) to yield 3*β*-lup-20(29)-en-ol (lupeol, **1**, 87.8 mg) and by re-crystallization with ethanol 96% yielded 3*β*-lup-20(29)-en-3-yl acetate (**2**, 592.3 mg). Gas chromatography (GC) and thin layer chromatography (TLC) analysis of the methanol extract indicated dulcitol as the primary constituent in the complex mixture which also contained phenolic compounds. The methanol extract (1.0 g) was acetylated [19] and partitioned into aqueous solution with ethyl acetate furnishing the dulcitol derivative 1,2,3,4,5,6-hexa-*O*-acetyldulcitol or dulcitol hexaacetate, **3** (400 mg). The structure elucidations of compounds **1**, **2** and **3** (Figure 1) from *M. acanthophylla* was done by spectroscopic (IR-FT, 1D and 2D Nuclear Magnetic Resonance) and spectrometric data (MS/EI) and by comparison with literature data previously published [20,21,22]. 

##### 3.1.3.3. Extraction and Isolation from *Xylosma flexuosa*

Air-dried aerial parts of *Xylosma flexuosa* (517 g) were ground into a powder and extracted exhaustively by maceration with *n*-hexane (1.5 L for three days) at room temperature. After filtration, the extract was concentrated *in vacuo* to yield 4.3 g of residue. The active *n*-hexane extract bioactivity measured on photophosphorylation showing an I_50_ (concentration producing 50% inhibition) of 79 ppm was subjected to column chromatography over silica gel (45 g) and eluted with a gradient of *n*-hexane-AcOEt. Four hundred and nineteen fractions (100 mL each) were collected and pooled on the basis of their TLC profiles to yield twelve major fractions (F1–F12). Bioactivities on the ATP synthesis for fractions F2, F3, F4, F5, F6, F9, 10, and F11 were tested; these fractions were obtained in enough amounts for the test and showed three active pools (F3, F4, and F10). F4 spontaneously precipitated (5 mg) as white crystals which were purified by crystallization with analytical reagent grade acetone (0.0562 g). The structure was established by its physical, spectroscopic (UV, IR, ^1^H- and ^13^C-NMR experiments), and spectrometric (GC-MS) data to afford the triterpene lup-20(29)-en-3β-ol (lupeol) [23]. F3 and F10 were obtained in small amounts and contain a mixture of compounds and were not further studied. The characterization of lupeol isolated from *Xylosma flexuosa* was carried out by comparison with the spectroscopic data from the literature [20].

Lup-20(29)-en-3*β*-ol (**1**): White crystalline powder (needles). M.p. 214–216 °C. IR-FT (KBr): 3550, 3400, 3295, 2920, 2850, 1640 (weak), 1455, 1380, 1040, 1015, 880. ^1^H-NMR (400 MHz; CDCl_3_) *δ*: 4.69 (br. d, 2.5 Hz, H-29b), 4,57 (dt, 2.5, 2.5 e 1.3 Hz, H-29a), 3.19 (dd, 11.0 e 5.0 Hz, H-3), 2.38 (dt, 11.0 e 5.8 Hz, H-19), 1.68 (br. dd, 1.3 e 0.8 Hz, H-30), 1.03 (s, H-26), 0.97 (s, H-23), 0.94 (br. d, 0.8 Hz, H-27), 0.83 (br. d, 0.8 Hz, H-25), 0.79 (s, H-28), 0.76 (s, H-24). ^13^C-NMR (100 MHz; CDCl_3_) *δ*: 0.96 (C-20), 109.32 (C-29), 79.01 (C-3), 55.32 (C-5), 50.46 (C-9), 48.33 (C-18), 47.78 (C-19), 43.01 (C-17), 42.85 (C-14), 40.85 (C-8), 40.02 (C-22), 38.87 (C-4), 38.73 (C-1), 38.07 (C-13), 37.19 (C-10), 35.60 (C-16), 34.30 (C-7), 29.87 (C-21), 28.00 (C-23), 27.46 (C-15), 27.43 (C-2), 25.17 (C-12), 20.95 (C-11), 19.32 (C-30), 18.33 (C-6), 18.01 (C-28), 16.12 (C-25), 15.99 (C-26), 15.37 (C-24), 14.56 (C-27). MS, *m/z* (%): 69 (100), 55 (72), 41 (64), 135 (52), 133 (34), 197 (28), 203 (26), 218 (26), 121 (44), 189 (40), 409 (1), 408 (1), 426 (1, M^+^, C_30_H_50_O).

3*β*-Lup-20(29)-en-3-yl acetate (**2**): White powder. M.p. 159–162 °C. IR-FT (KBr): 3070 (weak), 2920, 2850, 1735, 1640 (weak), 1455, 1380, 1370, 1250, 1010, 980, 880. ^1^H-NMR (400 MHz; CDCl_3_) *δ*: 4.69 (br. d, 3.0 Hz, H-29b), 4,57 (br. dt, 1.5, 1.5 e 0.8 Hz, H-29a), 4.47 (m, H-3), 2.37 (dt, 11.0 e 5.8 Hz, H-19), 2.04 (s, H-2′), 1.68 (br. s, H-30), 1.03 (s, H-26), 0.94 (s, H-27), 0.86 (s, H-25), 0.85 (s, H-23), 0.84 (s, H-24), 0.79 (s, H-28). ^13^C-NMR (100 MHz; CDCl_3_) *δ*: 171.01 (C-1′), 150.97 (C-20), 109.36 (C-29), 81,00 (C-3), 55.41 (C-5), 50.38 (C-9), 48.32 (C-18), 48.03 (C-19), 43.02 (C-17), 42.85 (C-14), 40.88 (C-8), 40.02 (C-22), 37.82 (C-4), 38.42 (C-1), 38.08 (C-13), 37.11 (C-10), 35.60 (C-16), 34.25 (C-7), 29.87 (C-21), 27.09 (C-23), 27.46 (C-15), 27.73 (C-2), 25.14 (C-12), 21,32 (C-2′), 20.97 (C-11), 19.31 (C-30), 18.23 (C-6), 18.02 (C-28), 16.51 (C-25), 16.00 (C-26), 16.19 (C-24), 14.52 (C-27). MS, *m/z* (%): 73 (100), 109 (55), 133 (34), 197 (28), 203 (23), 218 (15), 191 (44), 189 (44), 409 (1), 408 (2), 426 (4), 468 (3, M^+^, C_32_H_52_O_2_).

1,2,3,4,5,6-Hexa-*O*-acetyldulcitol (**3**): White powder. M.p. 168–170 °C. IR-FT (KBr): 3459, 2969 and 2955 (weak), 1747, 1376, 1247, 1227, 1084, 1053, 963, 608. ^1^H-NMR (200 MHz; CDCl_3_) *δ*: 5.36 (m, H-3 and H-4), 5.30 (m, H-2 and H-5), 4.28 (dd, 11.4, 4.4 Hz, H-1a and H-6a), 3.84 (dd, 11.4, 7.7 Hz, H-1b and H-6b), 2.11 (s, H-2′ and H-12′), 2.08 (s, H-6′ and H-8′), 2.02 (s, H-4′ and H-10’). ^13^C-NMR (50 MHz; CDCl_3_) *δ*: 170.46 (C-1′ and C-11′), 170.30 (C-3′ and C-9′), 169.78 (C-5′ and C-7′), 67.54 (C-2 and C-5), 67.45 (C-3 and C-4), 62.19 (C-1 and C-6), 20.72 (C-2′ and C-12′), 20.65 (C-4′ and C-10′), 20.58 (C-6′ and C-8′). MS, *m/z* (%):115 (100), 85 (46), 139 (52), 127 (42), 187 (43), 145 (33), 289 (25), 259 (16), 361 (6), 371 (5), 434 (2, M^+^, C_18_H_26_O_12_).

##### 3.1.3.4. Chloroplast Isolation and Chlorophyll Determination

Intact chloroplasts were obtained from spinach leaves (*Spinacea* *oleraceae* L) purchased from the local market as previously described [9,16] were suspended in a small volume of the following solution: 400 mM sucrose, 5 mM MgCl_2_, 10 mM KCl, and 30 mM of the buffer tricine-KOH (pH 8.0). They were stored as a concentrated suspension in the dark for 1 h at 4 °C. The chlorophyll (Chl) concentration was measured according to Strain [24].

##### 3.1.3.5. ATP Synthesis and Electron Flow Determinations

ATP synthesis was determined titrimetrically using a microelectrode (Orion model 8103; Ross, Beverly, MA, USA) connected to a Corning potentiometer model 12 (Corning Medical, Acton, MA, USA), with an expanded scale and a Gilson recorder (Kipp & Zonen, Bohemia, NY, USA) as previously reported [25]. Intact chloroplasts (20 μg Chl/mL) were broken before each assay by osmotic rupture in 3 mL of the non-buffered solution containing 100 mM sorbitol, 10 mM KCl, 5 mM MgCl_2_, 0.5 mM KCN, and 1 mM tricine-KOH at pH 8.0 in the presence of 50 μM methyl viologen (MV) and 1 mM adenosine triphosphate (ADP) at pH 6.7, and the pH was adjusted to 8.0 with 50 mM KOH. Alkalization rates were measured in the linear part during illumination. The reaction was calibrated by back-titration with saturated HCl and the ATP formed (μmol ATP mg^–1^·Chl h^–1^) was measured. Compounds at concentrations of 50, 100, 150, 200 and 300 μmol were prepared from 20 mM of stock solution, compounds were dissolved in dimethyl sulfoxide (DMSO).

##### 3.1.3.6. Measurements of Non-Cyclic Electron Transport Rate

Light-induced non-cyclic electron transport activity from water to MV, was performed using a Clark type electrode connected to an Oxygraph YSI (Yellow Spring Instrument) Model 5300. Basal electron transport was determined by illuminating chloroplasts (20 μg Chl/mL) during 1 min in 3 mL of medium containing 100 mM sorbitol, 10 mM KCl, 5 mM MgCl_2_, 0.5 mM KCN, 50 μM MV and 15 mM tricine-KOH (pH 8.0) as previously described [9,16]. Phosphorylating non-cyclic electron transport rate was measured as basal electron transport from water to MV except that 1 mM ADP and 3 mM KH_2_PO_4_ were added. Uncoupled electron transport was tested in the medium for basal electron transport, and 6 mM NH_4_Cl was added as uncoupler.

##### 3.1.3.7. Mg^2+^-ATPase Activity Assays

Chloroplasts were isolated from 30–40 g of spinach leaves, which were ground in 160 mL of medium containing 350 mM sorbitol, 5 mM ascorbic acid and 20 mM 2-(*N*-morpholino)-ethanesulfonic acid (MES) at pH 6.5. Chloroplasts were centrifuged at 3,000 *g* for 60 s, washed once in 40 mL of grinding medium, and re-suspended in 35 mM *N*-[2-hydroxyethyl]piperazine-*N′*-[2-ethanesulfonic acid] (HEPES) buffer (pH 7.6). Light-triggered Mg^2+^-ATPase activity associated to thylakoid membranes was measured as previously described [26], and released inorganic P was measured as reported [27].

##### 3.1.3.8. Ca^2+^-ATPase Activity Assays

Chloroplasts were diluted with 0.75 mM EDTA pH 7.8 to a final concentration of 0.4 mg of chlorophyll per mL and allowed to sit for 10 min at room temperature. CF_1_ depleted membranes were then removed by centrifugation. This EDTA extract (0.5 mL) was added to a 20 mM tricine pH 8.0, 2 mM EDTA, 10 mM DTT, 40 mM ATP (0.5 mL), heated at 60 °C for 4 min. and 0.1 mL of this activated mixture was incubated for 20 min at 37 °C with 0.9 mL of a medium containing 50 mM Tris pH 8.4, 5 mM ATP [26]. Reaction was stopped with 2% TCA and determination of Pi was evaluated [27]. In this case three repetitions were tested.

##### 3.1.3.9. Chlorophyll a Fluorescence Measurements in Spinach Leaf Discs

Chlorophyll *a* fluorescence was measured at room temperature with a Hansatech Fluorescence Handy PEA (plant efficiency analyzer) in dark-adapted spinach leaf discs (the physiological state of the leaf discs as dark adapted and standardized to kept the discs for 12 h in dark and light conditions before incubation with the compounds for 30 min in the dark using red light intensity (broad band 650 nm) of 3,000 µmol m^–2^ s^–1^, provided by an array of three light emitting diodes. The pulse duration was 2 s. Fifteen spinach leaf discs of 7 mm were placed in each Petri dishes with 10 mL of modified Krebs solution which contained: 115 mM NaCl, 5.9 mM KCl, 1.2 mM MgCl_2_, 1.2 mM KH_2_PO_4_, 1.2 mM Na_2_SO_4_, 2.5 mM CaCl_2_ and 25 mM NaHCO_3_ (pH = 7.4), the Petri dish were incubated for 12 h at room temperature. After this time for each Petri dish control DMSO was added in equal quantity that added to leaf disk treated with compounds. After this treatment, the leaf discs were dark adapted 30 min and immediately the chlorophyll fluorescence was measured. The OJIP transients were analyzed according to the JIP test. From the OJIP transient, the measured parameters were: Fluorescence intensity level at 50 μs when plastoquinone electron acceptor pool (Q_A_) is fully oxidized (F_0_); Fluorescence level when Q_A_ is transiently fully reduced (F_m_); variable component of fluorescence obtained by subtraction of F_0_ from the F_m_ value (F_V_). Different technical fluorescence photosynthetic parameters associated to PSII were obtained according to the equations of the O-J-I-P test [28,29,30]: (1) Derived parameters: V_J_ = (F_2ms_ − F_0_)/(F_m_ − F_0_), relative variable fluorescence at the J step (2 ms); Vi = (F_30ms_ − F_0_)/(F_m_ − F_0_), relative variable fluorescence at the I step; dV/dt_0_ = Mo = 4(F_300µs_ − F_0_)/(F_m_ − F_0_), approximated initial slope (in ms^−1^) of the fluorescence transient V = f(t); (2) Specific energy fluxes (per Q_A_^−^ reducing PSII center, RC): ABS/RC = (dV/dt_0_)(1/V_J_)(1/PHI(P_0_), Absorption flux per reaction center; TR_0_/RC = (dV/dt_0_)(1/V_J_), trapped energy flux per RC (at t = 0); ET_0_/RC = (dV/dt_0_)(1/V_J_)PHI(P_0_), electron transport flux per RC (at t = 0); (3) Phenomenological energy fluxes per excited cross section (CS): ABS/CS_0_ = F_0_, absorption flux per CS, approximated by F_0_; TR_0_/CS_0_ = (ABS/CS_0_) [PHI(P_0_)], trapped energy flux per excited cross section (CS) at t = 0; ET_0_/CS_0_ = (ABS/CS_0_) [PHI(E_0_)], electron transport flux per excited CS; (4) Yields or flux ratios: PHI(P_0_) = TR_0_/ABS = 1 − F_0_/F_m_, maximum quantum yield of primary photochemistry at t = 0; PSI_0_ = ET_0_/TR_0_ = (1 − VJ), probability at t = 0, that a trapped exciton moves an electron into the electron transport chain beyond Q_A_^−^; PHI(E_0_) = ET_0_/ABS = [1 − (F_0_/F_m_)], quantum yield of electron transport; PHI(D_0_) = 1 − PHI(P_0_) = F0/Fm, quantum yield (at t = 0) of energy dissipation; dR/RC = RE_0_/ET_0_ = (1− V_I_)/(1 − V_J_). W is the relative variable fluorescence between F_0_ and F_m_ calculated as V_t_ = F_Vt_/(F_m_ − F_0_) = (F_t_ − F_0_)/(F_m_ − F_0_) and plotted. K-band is calculated from W_OJ_, it is the relative variable fluorescence between F_0_ and F_J_ and V_OJ(__t)_ = F_Vt_/(F_J_ − F_0_) = (F_t_ − F_0_)/(F_J_ − F_0_) [30].

##### 3.1.3.10. Plant Material for *in Vivo* Assays

The seeds of *Physalis ixocarpa* and *Lolium perenne*, a weed species, were sown in 12 cm diameter pots and were watered daily in the greenhouse at 25 to 30 °C. After 15 and 18 days of emergence for *P. ixocarpa* and *L. perenne*, respectively, the plants were selected for similar size and were sprayed manually with the compounds **1** and **3** at concentrations of 150 and 300 µM (one stock of 20 mM of each compound was prepared in DMSO). An aliquot of this solution was taken to obtain the desired concentration in an aqueous suspension containing 0.05% w/v of polyoxyethylene sorbitan monolaurate . The control group was sprayed with distilled water containing the same amount of DMSO and Tween-20.

##### 3.1.3.11. Chlorophyll a Fluorescence Determination in Intact Leaves *in Vivo*

This was performed at room temperature with a portable Handy PEA apparatus [31], from the dark adapted for 15 min leaves, of control and treated plants, 24, 40 and 72 h after spraying with the compounds.

## 4. Conclusions

In conclusion, compound **1** interacts with H^+^-ATPase in thylakoids and acts as an energy coupling inhibitor when incubated for one minute. Further incubation time (7 min) of thylakoids with compound increases the inhibition activity. Furthermore, the fluorescence of chlorophyll *a* measured on spinach leaf disc incubated with **1** for 12 h induces the appearance of “heat sink centers” or silent reaction centers unable to reduce Q_A_, thus **1** inhibits donor site of PSII. Moreover, in *P. ixocarpa* leaves *in vivo* 150 µM **1** induced the appearance of the K band only at 48, and 72 h after treatment, thus interacting at the water splitting enzyme too. In the other hand, compound **3** acts as a Hill reaction inhibitor on thylakoids as determined by oxygen evolution, and when its activity was measured on spinach leaf disc by fluorescence, it induced the appearance of the K band indicating that it inhibits the water splitting enzyme. When *P. ixocarpa* plants were sprayed with **3** it induces the appearance K and I bands after 72 h of treatment indicating two targets of interaction, one at the OEC complex and the second at the PQH_2_ (plastoquinone pool) site, the b_6_f complex.
